# A novel method for computing state transition matrices due to the unscented transform

**DOI:** 10.1007/s10569-025-10247-1

**Published:** 2025-04-28

**Authors:** Rahil Makadia, Davide Farnocchia, Steven R. Chesley, Siegfried Eggl

**Affiliations:** 1 Department of Aerospace Engineering, University of Illinois at Urbana-Champaign, 104 S. Wright St., Urbana, IL 61801 USA; 2 Jet Propulsion Laboratory, California Institute of Technology, 4800 Oak Grove Dr., Pasadena, CA 91109 USA

**Keywords:** Dynamical systems theory, Celestial mechanics, Entry flight mechanics, Unscented transform

## Abstract

We present a new method for computing the state transition matrix of a nonlinear dynamical system. The proposed method does not require the implementation of complex partial derivatives or auto-differentiation of the dynamics, while removing the arbitrary choice of a perturbation step for traditional finite difference methods. We tested the new state transition matrices using three different applications: a simple two-body problem, a Mars atmospheric entry flight mechanics problem, and two future close encounters of the asteroid (101955) Bennu with the Earth. Results show that the unscented transform state transition matrices preserve symplecticity and perform just as well as the classical unscented transform. Furthermore, the new method can closely reproduce posterior distributions generated using Monte Carlo simulations, even in the presence of significant stiffness in the dynamics.

## Introduction

State transition matrices (STMs) are a fundamental tool in the field of dynamical systems theory. For linear systems, STMs can be computed analytically and provide an exact map of the state at any given time, provided an initial state. However, for nonlinear systems, the computation of STMs is not as straightforward. In this case, the STM gives a map of how small deviations in the initial state evolve over time, and the accuracy of the STM depends on the size of the initial deviation and the duration over which it is mapped. This is particularly important in the field of astrodynamics and celestial mechanics, where the nonlinear nature of the equations of motion makes the computation of STMs a challenging task.

Traditionally, STMs have been calculated using one of two methods. The first method involves linearizing the equations of motion about a nominal trajectory and integrating the variational equations to solve for the STM (Milani and Gronchi 2009, Ch. 2). This method has been used for decades in operational orbit determination routines and is still widely used today. However, solving the variational equations is not straightforward. This method involves the derivation of the analytic partial derivatives of the dynamical model, which adds an additional layer of complexity. The second method involves numerically computing the derivatives of the dynamics using finite differences. This second method is more straightforward than the analytic partial derivatives, but it is also more expensive in terms of computational cost. Furthermore, it requires the user to manually select a suitable step size for the finite difference perturbations. This is subject to accuracy limitations due to the interplay between truncation errors and floating-point precision (Rein and Tamayo 2016).

More recently, several new methods have been developed to compute STMs for nonlinear systems. These methods include using higher-order state transition tensors (Park and Scheeres 2006), polynomial chaos expansions (Jones et al. 2013), differential algebra (Armellin et al. 2010), and automatic differentiation (Bani Younes 2019). However, these methods have their own drawbacks. For example, higher-order state transition tensors require computing the solution of their governing equations, which can be computationally expensive. Polynomial chaos expansions and differential algebra are good alternatives to massive Monte Carlo simulations, but the computational cost can still be too high when looking to compute the STM (as opposed to computing just the posterior distributions). Automatic differentiation is a powerful tool that can be used to compute the STM, but it requires access to the source code of the dynamics (which is also the case for differential algebra), which is not always possible. Even when this is possible, it requires an external reimplementation of basic mathematical operations in the automatic differentiation framework, which is not always an option.

In this paper, we present a method for computing STMs using the unscented transform and apply it to various problems in celestial and spaceflight mechanics. Compared to analytic STMs, the proposed method does not require the implementation of complex partial derivatives or automatic differentiation of the dynamics. It also alleviates the problem of finite differences not working in all situations of the same problem (e.g., orbit determination for near-Earth asteroids as opposed to main-belt asteroids). In terms of performance, the unscented transform STMs evaluate the same number of trajectories as second-order central differences, so the computational cost is no worse than traditional finite difference STMs. Results also show that the accuracy of these new unscented STMs is similar to or better than the finite difference STMs.

## Methods

### Sigma points and the unscented transform

The unscented transform was first introduced by Jeffrey Uhlmann to improve filtering in cases where the extended Kalman filter (EKF) does not perform well (Uhlmann 1995). The primary application of this work was for filtering a set of observations for the state and any dynamical parameters in a nonlinear system. Uhlmann’s original work yielded the unscented Kalman filter (UKF). The first step in the unscented transform procedure is to select a set of sigma points. These (usually $2n+1$, where *n* is the dimension of the system) sigma points are chosen such that they are symmetric about the mean and are correctly scaled in order to preserve the covariance of the initial estimate. Various formulations have been used to generate sigma points, differing in the weights assigned to each point (Julier and Uhlmann 1997; van der Merwe 2004). These symmetric sigma points and weights can be generalized as shown below:

1 $$\begin{aligned} &  \varvec{{\mathcal {X}}_0} = \overline{{\textbf{x}}} \nonumber \\ &  \varvec{{\mathcal {X}}_i} = \overline{{\textbf{x}}}+\left( \sqrt{(n+\kappa ) {\textbf{P}}_{x x}}\right) _i \end{aligned}$$

2 $$\begin{aligned} &  \varvec{{\mathcal {X}}_{i+n}} = \overline{{\textbf{x}}}-\left( \sqrt{(n+\kappa ) {\textbf{P}}_{x x}}\right) _i \end{aligned}$$

Here, $\varvec{{\mathcal {X}}}$ is the $(2n+1) \times n$ array of sigma points, $\overline{{\textbf{x}}}$ is the mean of the initial state, ${\textbf{P}}_{x x}$ is the covariance of the initial state, and $\kappa$ is a scaling factor. $W_0=\kappa /(n+\kappa )$ is the weight assigned to the mean, and $W_i = W_{i+n} = 0.5/(n+\kappa )$ are the weights assigned to the other sigma points. These weights correspond to the formulation given by Julier and Uhlmann (1997). In this formulation, $\kappa$ allows for some fine-tuning of the spread of the sigma points. In the case where $\kappa =0$, it reduces to the original Uhlmann sigma points. This is also the value for $\kappa$ adopted throughout this work.

The sigma points are then passed through the nonlinear transformation to obtain the transformed sigma points, $\varvec{{\mathcal {Y}}_i}={\textbf{f}}\left[ \varvec{{\mathcal {X}}}_i\right]$. The mean and covariance of the transformed sigma points are then computed as their respective weighted operations:

3 $$\begin{aligned} \begin{aligned} \overline{{\textbf{y}}}&=\sum _{i=0}^{2 n} W_i \varvec{{\mathcal {Y}}_i} \\ {\textbf{P}}&=\sum _{i=0}^{2 n} W_i\left\{ \varvec{{\mathcal {Y}}_i}-\overline{{\textbf{y}}}\right\} \left\{ \varvec{{\mathcal {Y}}_i}-\overline{{\textbf{y}}}\right\} ^T. \end{aligned} \end{aligned}$$

As mentioned previously, this unscented transform has already been used in a Kalman filter application to improve the performance of the filter in the presence of nonlinearity, resulting in the unscented Kalman filter (UKF) (Wan and van der Merwe 2000; Julier and Uhlmann 2004).

**Fig. 1:**
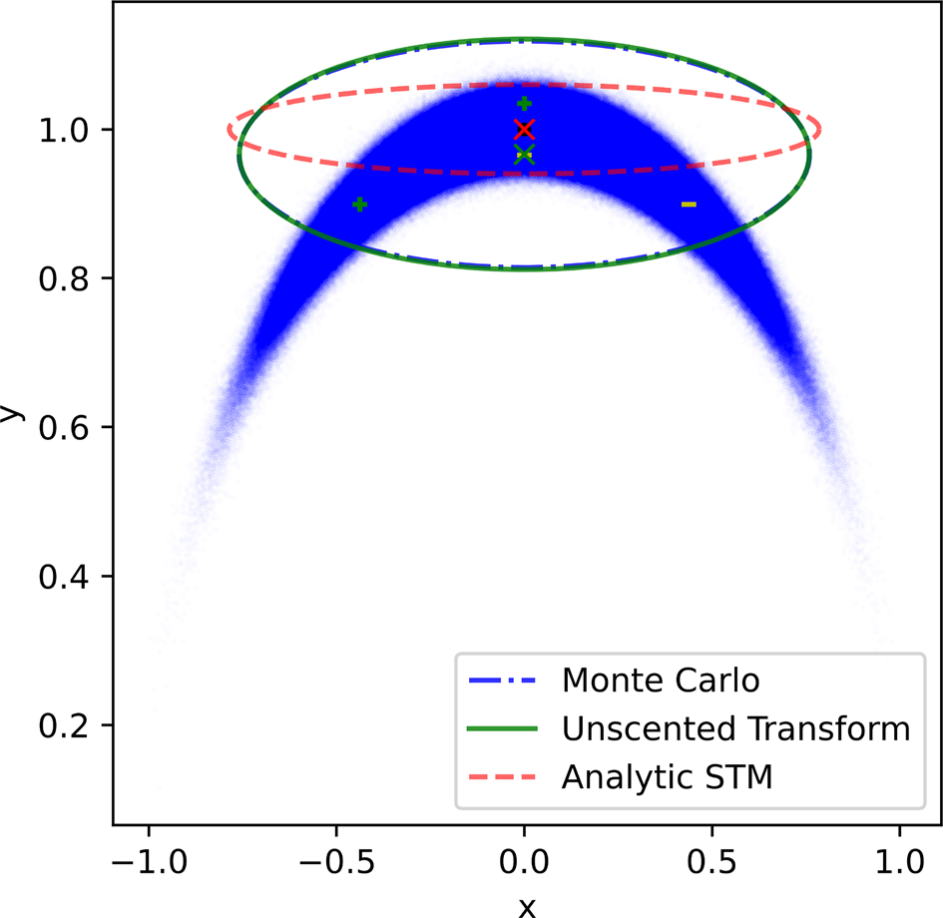
Monte Carlo samples and 3$\sigma$ uncertainty ellipses for a conversion from polar to Cartesian coordinates using a Monte Carlo simulation (using 10$^7$ samples, shown as a cloud of scatter points), the unscented transform (using 5 Sigma points), and an analytic STM. The crosses show the means (the Monte Carlo and sigma point mean coordinates are indistinguishable because they overlap). The black dot (which coincides with the analytic mean) corresponds to the transformed value of the nominal polar coordinates. The scatter points labeled ‘+’ are the positively perturbed sigma points from Equation (1), and the scatter points labeled ‘-’ are the negatively perturbed sigma points from Equation (2)

Figure 1 shows a demonstration of the unscented transform for a simple nonlinear function. The transformation simply converts polar coordinates to Cartesian coordinates. The polar coordinate distribution has a mean distance-angle pair of $\left[ 1,90^{\circ }\right]$ and a diagonal covariance with standard deviations of $2\times 10^{-2}$ and $15^{\circ }$. We see that the unscented transform (used in the UKF) is clearly more accurate than the analytic covariance transformation using the STM (used in the EKF) when the two are compared to a Monte Carlo sampling using 10 million samples.

Therefore, the UKF can outperform the EKF in terms of accuracy (especially in the presence of significant uncertainties). However, in its current form, the UKF directly reconstructs the posterior mean and covariance. It does not provide any information about the partial derivatives of the dynamics, which is encoded in the STM. This precludes the use of the unscented transform for algorithms where the STM is required, such as a least squares filter or a classical EKF instead of the UKF. This work aims to fill this gap and develop methods to compute the STM of any given nonlinear transformation using the unscented transform.

### Unscented transform STMs

For nonlinear dynamical systems, the STM ($\mathbf {\Phi }$) maps small deviations of the nominal state from an initial time ($\delta {\textbf{x}}_0$) to the corresponding deviations at a final time ($\delta {\textbf{x}}_f$), i.e.,

4 $$\begin{aligned} \delta {\textbf{x}}_f = \mathbf {\Phi } \delta {\textbf{x}}_0. \end{aligned}$$

The STM can produce linearized estimates for the evolution of the state and covariance of a nonlinear dynamical system. As a result of this, it has been a key component in estimation algorithms for decades (Kalman 1960).

The proposed method relies on the fact that the unscented transform uses sigma points, which are a symmetric set of points around the initial mean state. As shown by Equations (1) and (2), there is an $n \times n$ array of initial sigma points that are perturbed from the mean in the positive direction ($\delta {\textbf{x}}_0^+$), and another array that is perturbed in the negative direction ($\delta {\textbf{x}}_0^-$).

Therefore, the numerical propagation of the sigma points is equivalent to propagating small perturbations to the initial state. The mapping of small deviations of the nominal state from an initial time to a final time is exactly what the STM does. The positively perturbed sigma points at the final time therefore give us $\delta {\textbf{x}}_f^+$, and correspondingly, the negative ones give us $\delta {\textbf{x}}_f^-$. These two matrices allow us to solve for the STM using the state mapping relationship from Eq. 4:

5 $$\begin{aligned} \begin{aligned} \mathbf {\Phi }^+&= \delta {\textbf{x}}_f^+ (\delta {\textbf{x}}_0^+)^{-1} \\ \mathbf {\Phi }^-&= \delta {\textbf{x}}_f^- (\delta {\textbf{x}}_0^-)^{-1} \end{aligned} \end{aligned}$$

where $\mathbf {\Phi }^+$ and $\mathbf {\Phi }^-$ are the STMs for the positively and negatively perturbed sigma points, respectively. Given that equal weights are assigned to the positively and negatively perturbed sigma points when reconstructing the posterior mean and covariance, the unscented transform STM can simply be computed as the average of the positively and negatively perturbed STMs:

6 $$\begin{aligned} \mathbf {\Phi }^{UT} = \frac{1}{2} \left( \mathbf {\Phi }^+ + \mathbf {\Phi }^-\right) . \end{aligned}$$

## Results and discussion

In order to validate and demonstrate the performance of these unscented transform STMs, we present three different applications. The first is a simple two-body problem, where the equations of motion are simpler and the dynamics are usually not stiff. The second example involves application to a Mars atmospheric entry flight mechanics problem, where the equations of motion are more complex but there are no significant nonlinearities that make the dynamics stiff during integration. The third case study involves the mapping of the B-plane covariances for an asteroid with deep planetary encounters, where the equations of motion are of operationally high fidelity and there is significant stiffness introduced to the system by the repeated close approaches.

### Two-body problem

In order to fully test the unscented transform STMs, we first consider a classical two-body problem. This allows us to compare the unscented transform STMs to the analytic STMs and perform some mathematical checks on both STMs. This ensures that the unscented transform STMs are valid and accurate. The equations of motion for the two-body problem are given by:

7 $$\begin{aligned} \begin{aligned} {\textbf{x}}&= \begin{bmatrix} {\textbf{r}} \\ {\dot{\textbf{r}}} \end{bmatrix} \\ \dot{{\textbf{x}}}&= f({\textbf{x}}) = \begin{bmatrix} {\dot{\textbf{r}}} \\ \ddot{\textbf{r}} \end{bmatrix} \\ \ddot{\textbf{r}}&= -\frac{\mu }{r^3} {\textbf{r}} \end{aligned} \end{aligned}$$

where ${\textbf{r}}$ is the relative position vector, ${\dot{\textbf{r}}}$ is the relative velocity vector, and $\mu$ is the gravitational parameter. Additionally, if propagating the STM, we add the STM dynamics to the system, which can be written as:

8 $$\begin{aligned} \begin{aligned} \dot{\varvec{\Phi }}&= {\textbf{A}} \varvec{\Phi } \\ {\textbf{A}}&= \frac{\partial f}{\partial {\textbf{x}}} = \begin{bmatrix} {\textbf{0}}_{3\times 3} & {\mathbb {I}}_{3\times 3} \\ -\frac{\mu }{r^3} {\mathbb {I}}_{3\times 3} + 3\frac{\mu }{r^5} {\textbf{r}}\otimes {\textbf{r}} & {\textbf{0}}_{3\times 3} \end{bmatrix} \end{aligned} \end{aligned}$$

where $\varvec{\Phi }$ is the STM and ${\textbf{A}}$ is the partial derivative matrix that governs the dynamics of the analytic STM.

As a specific example, we consider the Sun–Earth system with the Earth in a simple circular orbit around the Sun. Therefore, the initial conditions in canonical units using Distance Units (DU, 1 DU = 1 au = $1.495 978 707 \times 10^{11}\hbox { m}$) and Time Units (TU, 1 TU = ${1/2\pi }{\hbox {yr}}$ = $5.021 897 736 \times 10^{6}{\hbox {s}}$) are given by:

9 $$\begin{aligned} {\textbf{r}}_0 = \begin{bmatrix} 1 \\ 0 \\ 0 \end{bmatrix} \quad {\dot{\textbf{r}}}_0 = \begin{bmatrix} 0 \\ 1 \\ 0 \end{bmatrix} \quad \varvec{\Phi }_0 = {\mathbb {I}}_{6\times 6}. \end{aligned}$$

We propagate these initial conditions for 1 orbit period ($2\pi$ TU) and compute the final state as well as the analytic, central difference, and unscented transform STMs. Additionally, we assume a diagonal covariance with position and velocity standard deviations of $1000\hbox { m}$ and $1 \times 10^{-3}\hbox {m s}^{-1}$, respectively. Propagating these uncertainties using the STMs gives the final covariance of the state after 1 orbit period. These covariances, combined with the STMs, can be used to perform a series of tests.

Since the two-body system is conservative, the STM should be symplectic. Therefore, we can perform some tests to ensure the validity of the STMs. The first test is to compute how well the STM preserves the symplectic condition

10 $$\begin{aligned} \varvec{\Phi }^T {\textbf{J}} \varvec{\Phi } - {\textbf{J}} = {\textbf{0}}, \end{aligned}$$

where ${\textbf{J}}$ is a non-singular, skew-symmetric matrix. A common choice is the matrix

11 $$\begin{aligned} {\textbf{J}} = \begin{bmatrix} {\textbf{0}}_{3\times 3} & {\mathbb {I}}_{3\times 3} \\ -{\mathbb {I}}_{3\times 3} & {\textbf{0}}_{3\times 3} \end{bmatrix}. \end{aligned}$$

Evaluating the left-hand side of Equation (10) for the analytic, central difference, and unscented transform STMs and taking the Frobenius norm (Golub and Loan 2013, Ch. 2) give us the results presented in the first row of Table 1. This discrepancy between the analytic STMs and the other two methods exists because the analytic STMs are integrated using the variational equations, which are symplectic by construction. Therefore, the resulting violation in symplectic behavior is simply a function of integration tolerance, and the metric has a value much closer to 0 The other two methods have higher errors because the unscented transform STMs and central difference STMs do not have a way to strictly preserve this symplectic condition. The magnitude of this metric for the unscented STM is still very close to zero, especially given that symplecticity is not formally enforced, and therefore not a cause for concern.

The second test is to confirm that the determinants of the STMs are equal to 1. This is because the STM should not change the volume of the state space taken up by the covariance ellipses. The results of this test for the three STMs are presented in the second row of Table 1. All values are very close to zero once again, and the expected behavior of the analytic STM being closer to zero is repeated as well. Therefore, the unscented transform STMs successfully preserve the symplectic condition.

Further tests included comparing the mapped covariances according to the formula $||{\textbf{P}} - {\textbf{P}}^{A}||_F/||{\textbf{P}}^{A}||_F$, where ${\textbf{P}}$ is either the covariance computed using the central difference STM or the unscented transform STM, ${\textbf{P}}^{A}$ is computed using the analytic STM, and $||\cdot ||_F$ is the Frobenius norm. The results of this test are shown in the third row of Table 1. Both the central difference STMs and unscented STMs have the same error, further validating the new unscented STMs. However, this comparison depends on the chosen initial covariance. To remove this dependence, we did the same comparison directly for the two STMs as $||\varvec{\Phi } - \varvec{\Phi }^{A}||_F/||\varvec{\Phi }^{A}||_F$. The value of this metric was $4\times 10^{-7}$ for the unscented STM and $6\times 10^{-6}$ for the central difference STM. These results show that the unscented transform STMs practically have the same accuracy as the analytic STMs for application to the two-body problem. The results from all four tests are summarized in Table 1.

**Table 1:** Summary of errors for the Sun–Earth 2-body problem tests using the analytic, central difference, and unscented transform STMs. Tests that use the analytic STM as ground truth do not have a corresponding value in the analytic STM column, and are marked ‘-’

Test	Analytic STM	Central difference STM	Unscented STM
Symplectic Check	$1\times 10^{-11}$	$2\times 10^{-4}$	$4\times 10^{-4}$
STM Determinant Check	$1\times 10^{-14}$	$1\times 10^{-5}$	$1\times 10^{-6}$
Mapped Covariance Errors	–	$9\times 10^{-7}$	$9\times 10^{-7}$
Direct STM Comparison	–	$6\times 10^{-6}$	$4\times 10^{-7}$

To further investigate the performance of the unscented STMs against analytic STMs in a two-body problem setting, we add an example using a representative main-belt asteroid with orbital elements as listed in Table 2.

**Table 2:** Initial orbital elements for a representative main-belt asteroid

Orbital Element	Value
Semimajor axis [DU]	3
Eccentricity	0-1 (varies)
Inclination [deg]	0
Right Ascension of the Ascending Node [deg]	0
Argument of Perihelion [deg]	0
Mean Anomaly [deg]	0

**Fig. 2:**
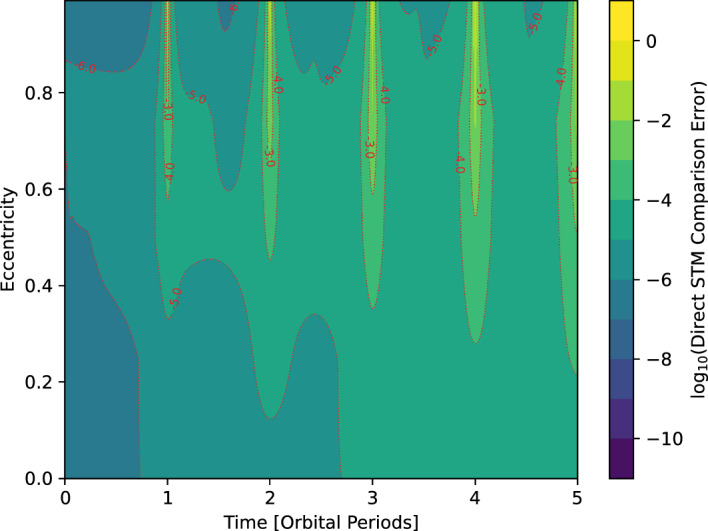
Direct comparison errors for the unscented STMs for a representative main-belt asteroid

For varying values of orbit eccentricity and integration span, we repeat the direct STM comparison test ($||\varvec{\Phi } - \varvec{\Phi }^{A}||_F/||\varvec{\Phi }^{A}||_F$) done in the Sun–Earth system above. The results for this test are shown in Fig. 2. As expected, the STM comparison errors increase as the propagation duration or eccentricity increases. The increase in error as a function of eccentricity is larger than the one corresponding to increasing orbital periods. Highly eccentric orbits have larger errors at each perihelion pass. This is to be expected, as the quality of numerical integration deteriorates during close encounters when the accelerations on the asteroid are rapidly changing. However, most of the errors are low, with the average error being less than 0.05% over the entire testing region of Fig. 2, further validating the reliability of the unscented STMs.

A natural question arises: If the STM is routinely used to map the covariance of the state, why not just use the existing unscented transform formalism to map the covariance directly? The answer is that the STM is a powerful tool that can do more than just map covariances. For example, in the context of the more complicated three-body problem, integrating the STM for one orbit period yields the monodromy matrix. The monodromy matrix can be used to study the stable and unstable manifolds of the system. These manifolds are a vital concept used to design spacecraft trajectories in the Earth–Moon system and beyond. To further evaluate the use of the unscented transform STMs, we will now consider applying them to more complex dynamics.

### Entry, descent, and landing flight mechanics

Realistic applications of spaceflight mechanics involve more complicated dynamics than the two-body problem. A consequential example of this is the problem of determining the landing site of a spacecraft entering the atmosphere of a planet. This problem is known as Entry, Descent, and Landing (EDL) and has been a critical part of many missions to Mars, such as the Mars Science Laboratory (MSL) vehicle that landed the Curiosity rover and the Mars 2020 vehicle that landed the Perseverance rover. EDL dynamics involve a variety of forces, including gravity, drag, and lift (and even thrust in many instances). Here, we perform trajectory simulations for an MSL-like spacecraft ballistically entering the Martian atmosphere.

**Table 3:** EDL simulation parameters used for the landing site dispersion analysis

*Vehicle parameters*	
Mass, m	3300 kg
Base Radius, r$_b$	2.25 m
Drag Coefficient, C$_D$	1.4
Lift Coefficient, C$_L$	0.34
Ballistic Coefficient, $\beta$	$148\hbox { kg/m}^{2}$
*State at entry interface*	
Radius, r	3522.2 km
Velocity, V	$6.0833\hbox { km s}^{-1}$
Flight Path Angle, $\gamma$	$-15.4892^{\circ }$
Azimuth, $\psi$	$93.2065^{\circ }$
Latitude, $\phi$	$-3.9186^{\circ }$
Longitude, $\theta$	$126.72^{\circ }$

The dynamics of this problem as well as the initial conditions (state and covariance) are taken from the MSL EDL reconstruction performed by Dutta and Braun (2014). The representative vehicle parameters are taken from Meginnis et al. (2013) and are listed along with the state at entry interface in Table 3. The equations of motion for the EDL problem are given by:

12 $$\begin{aligned} {\dot{r}}&=V \sin \gamma \nonumber \\ {\dot{V}}&=-\frac{F_D}{m}-g \sin \gamma +\omega ^2 r \cos \phi (\sin \gamma \cos \phi -\cos \gamma \sin \phi \sin \psi ) \nonumber \\ {\dot{\gamma }}&=\frac{1}{V}\left[ \frac{F_L \cos \nu }{m}-g \cos \gamma +\omega ^2 r \cos \phi (\cos \gamma \cos \phi +\sin \gamma \sin \phi \sin \psi ) \right. \nonumber \\&\qquad \quad \left. +\frac{V^2}{r} \cos \gamma +2 \omega V \cos \phi \cos \psi \right] \nonumber \\ {\dot{\psi }}&=\frac{1}{V}\left[ \frac{F_L \sin \nu }{m \cos \gamma }-\frac{V^2}{r} \cos \gamma \cos \psi \tan \phi \right. +2 \omega V(\tan \gamma \cos \phi \sin \psi -\sin \phi ) \nonumber \\&\qquad \quad \left. -\frac{\omega ^2 r}{\cos \gamma } \sin \phi \cos \phi \cos \psi \right] \nonumber \\ {\dot{\phi }}&=\frac{V \cos \gamma \sin \psi }{r} \nonumber \\ {\dot{\theta }}&=\frac{V \cos \gamma \cos \psi }{r \cos \phi } \end{aligned}$$

where *r* is the altitude, *V* is the velocity magnitude, $\gamma$ is the flight path angle, $\psi$ is the azimuth angle, $\phi$ is the latitude, and $\theta$ is the longitude of the vehicle. Furthermore, $\omega$ is the rotation rate of Mars and $\nu$ is the vehicle bank angle. The forces $F_D$ and $F_L$ are the drag and lift forces, respectively:

13 $$\begin{aligned} \begin{aligned} F_D&= \frac{1}{2} \rho V^2 C_D A \\ F_L&= \frac{1}{2} \rho V^2 C_L A \end{aligned} \end{aligned}$$

Here, $\rho$ is the atmospheric density, $C_D$ and $C_L$ are the drag and lift coefficients, and *A* is the reference area computed using the base radius of the vehicle. A simple exponential atmospheric model^1^ is used to compute the density because more complex models such as the Mars Global Reference Atmospheric Model (Mars-GRAM) (Justus et al. 2002) are not easily available.

**Fig. 3:**
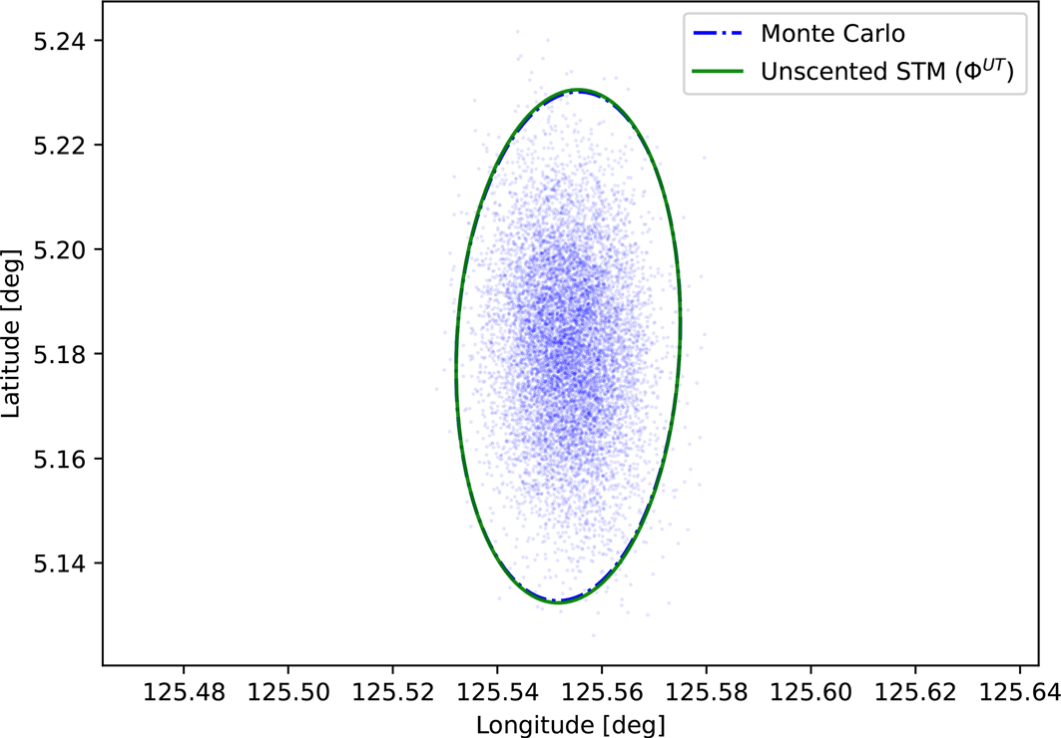
$3\sigma$ landing site dispersion ellipses for an example Mars EDL scenario. The scatter points show the landing site for 10$^4$ Monte Carlo samples, and the ellipses are computed using 2 different methods (Monte Carlo and unscented transform STM)

The equations of motion were integrated using the MATLAB ode89 routine. A Monte Carlo simulation with $10^4$ samples was performed to calculate the dispersion of the ‘true’ landing site. 13 sigma points were then propagated to obtain the unscented transform STM. The unscented transform STM was subsequently used to compute the covariance of the landing site coordinates. The results are shown in Fig. 3. The Monte Carlo samples are shown as scatter points, and the ellipses show the $3\sigma$ dispersion.

There is excellent agreement between the Monte Carlo and unscented transform ellipses. Using the Monte Carlo ellipse as a reference, the unscented transform ellipse had a difference of 0.79% in the semimajor axis, $-$0.04% in the semiminor axis, and 0.20% in the orientation angle of the ellipse with respect to the horizontal. Therefore, the unscented transform STMs are accurate for the EDL problem and can be used to map the covariance of the landing site.

As mentioned previously, the unscented transform naturally produces the posterior mean and covariance of the state. Therefore, it would be of perfect use in such an application. However, the STM has the added advantage of also being the Jacobian of the final state with respect to the initial state. In simulations with more complex EDL models (ones with more realistic uncertainties in atmospheric models or vehicle parameters), the STM can be used to compute the sensitivity of the landing site with respect to individual uncertain parameters. This is a powerful tool that can help with the design of the entry vehicle and landing site selection. Furthermore, the STMs computed using the unscented transform yield the same covariances as the unscented transform itself. In the example presented here, all three ellipse parameters (the semimajor axis, semiminor axis, and the orientation angle of the ellipse) agree to better than 0.00001%. This demonstrates that the unscented transform STMs are just as accurate as the unscented transform itself while providing more insight into the dynamics of the system.

### Earth close encounters of asteroid (101955) Bennu

Thus far, the unscented transform STMs have been shown to be accurate for both simple and complex dynamics. However, full validation of the unscented transform STMs can occur when the dynamics are highly nonlinear and stiffness is introduced to the system over time. In this case, we consider the asteroid (101955) Bennu, a near-Earth asteroid that has recurring close approaches with Earth. In this example, the unscented transform STMs are used to map the B-plane uncertainties of Bennu during select close encounters with the Earth.

The B-plane is an essential tool for designing interplanetary trajectories as well as studying planetary close encounters of asteroids to assess the potential of an impact (Farnocchia et al. 2019). It is a two-dimensional plane that contains the geocenter at the origin and is defined by the incoming hyperbolic asymptote of the asteroid. The B-plane contains the B-vector, which is the vector from the center of the Earth to the point where the hyperbolic asymptote intersects the plane. Traditionally, the points on a B-plane are described using the standard (B.R, B.T) basis, which is useful for studying the trajectories of interplanetary spacecraft (Kizner 1961). However, in the context of asteroid close encounters with the Earth, the Öpik formulation can be more useful (Farnocchia et al. 2019). The Öpik formulation uses the ($\xi , \zeta$) coordinates, which are a simple two-dimensional rotation of the (B.R, B.T) coordinates about the incoming hyperbolic asymptote. This representation is convenient because $|\xi |$ is the minimum orbit intersection distance of the asteroid with the Earth, and $\zeta$ provides information about the timing of the close encounter ($\zeta <0$ implies the asteroid arrives at the close encounter condition before the Earth, and $\zeta >0$ implies the asteroid arrives after the Earth).

High-accuracy modeling and prediction of the motion of asteroids calls for high-fidelity dynamical models. In this study, we use the following dynamical model for propagating an asteroid in the solar system:

- Newtonian point-mass gravity of the Sun, planets, Pluto, the Moon, and the 16 largest main-belt asteroids.
- Einstein–Infeld–Hoffmann (EIH) relativistic corrections to the Newtonian gravity of the Sun, planets, Pluto, and the Moon.
- 2^nd^-order zonal harmonics (J_2_) of the Sun and Earth.
- Radial and transverse non-gravitational accelerations due to solar radiation pressure as well as the Yarkovsky effect.

Although the STM is integrated in the same manner as Equation (8), the partial derivative matrix ${\textbf{A}}$ is significantly more complex due to the detailed force model used to calculate the accelerations on the asteroid. However, it is still possible to analytically compute the STM for this system. In this test scenario, we compare the unscented transform STMs to those computed using this analytic method as well as STMs computed numerically using central finite difference methods. To remain consistent for all STM computations, we only use the STM of the Cartesian state at close approach time and then convert it to the covariance of the B-plane coordinates using the partial derivative formulation described in Farnocchia et al. (2019). Due to the significant nonlinearities in the dynamics of (101955) Bennu, we also run a massive Monte Carlo simulation using $10^6$ samples of the initial covariance to serve as ground truth for evaluating the accuracy of the different STM calculation methods.

We used the Bennu observation data arc used by Chesley et al. (2014) as the starting point for this analysis (September 1999–January 2013). It is important to note that a more comprehensive analysis of future Bennu impact probabilities was performed by Farnocchia et al. (2021). This updated work made use of astrometry from the OSIRIS-REx spacecraft at Bennu, which required unprecedented dynamical models to predict the asteroid’s trajectory. In order to keep the focus on demonstrating the performance of the unscented transform STMs rather than the dynamical models used to predict asteroid trajectories, we use the preceding orbit solution from Chesley et al. (2014).

Using the same observations as Chesley et al. (2014), we compute an orbit solution using the GRSS library^2^. The dynamical and observational models used in this library have been extensively tested with operational asteroid monitoring programs used at JPL and should be considered fully validated (Makadia et al. 2024, 2025). We also use the same library to propagate the Monte Carlo samples until the end of 2135 and compute the STMs using the different methods. The simulation end time of 31 December 2135 is chosen because during the 2135 close encounter of Bennu with the Earth, the asteroid is significantly perturbed. As a result, Bennu’s trajectory after this encounter is no longer deterministic.

**Fig. 4:**
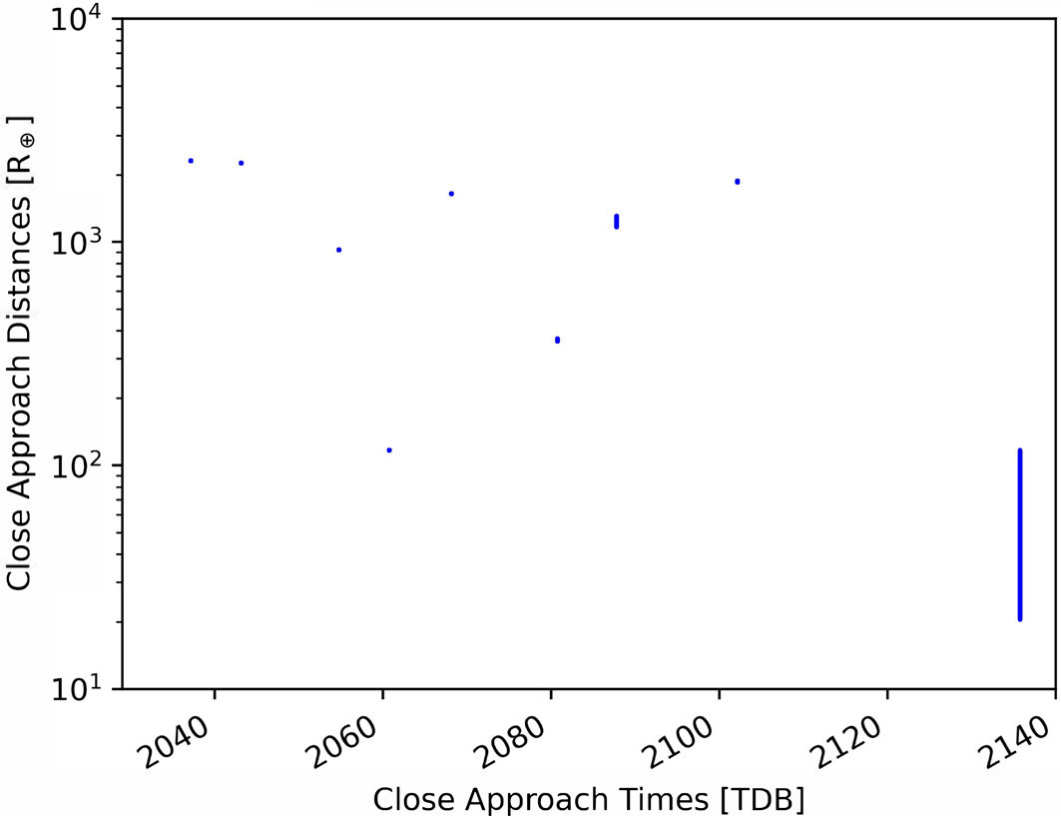
Earth close approach distances (in units of Earth radii) from $10^6$ Monte Carlo samples of Bennu until the year 2135. The uncertainty in close approach distances starts increasing after the 2080 encounter. Note the logarithmic scale for the ordinate

Figure 4 shows the close approach distances of the Monte Carlo samples until 2135. The spread of these distances increases significantly after the 2080 Earth encounter and grows to tens of thousands of kilometers in 2135. This is due to the deep planetary encounters that Bennu experiences, which significantly perturb the asteroid’s trajectory. In order to evaluate the accuracy of the different STMs in varying levels of nonlinearity, we pick the 2080 (when the uncertainties are relatively small) and 2135 (when the uncertainties are large) encounters to compare the B-plane maps.

**Fig. 5:**
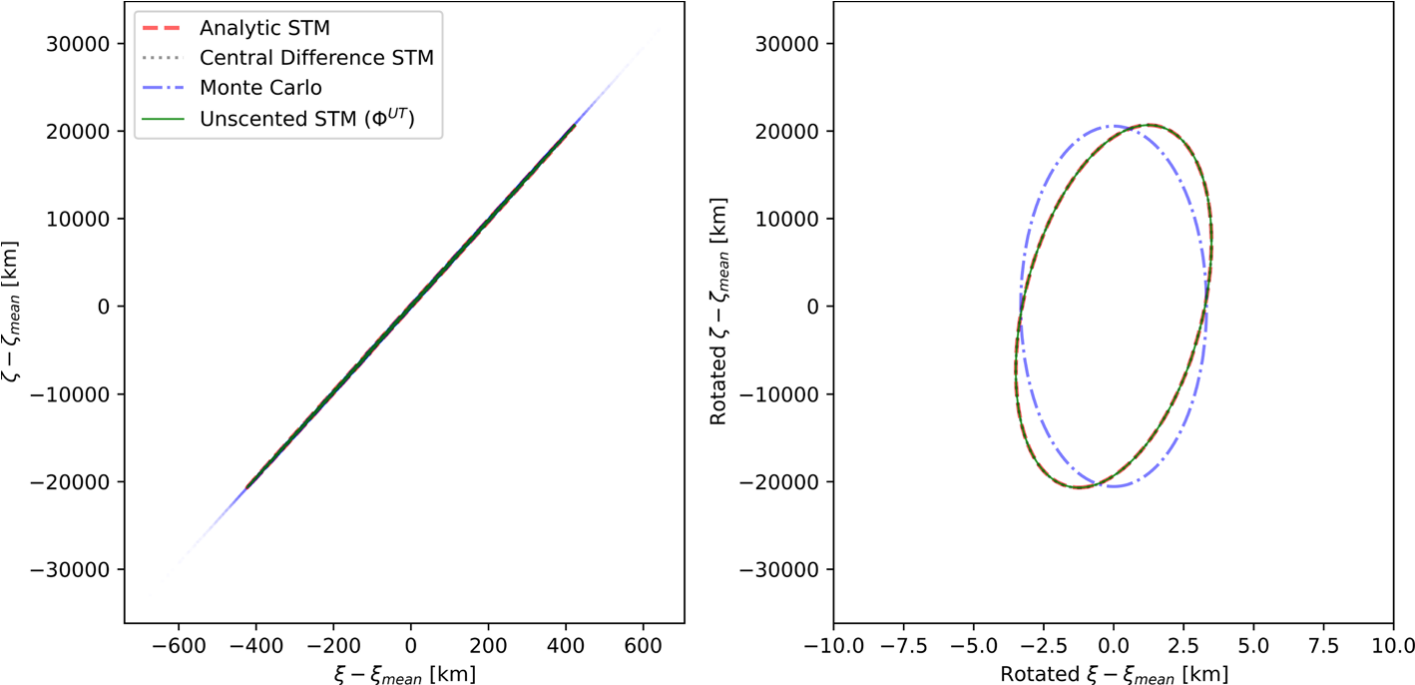
3$\sigma$ ellipses of the B-plane uncertainties for Bennu during the 2080 Earth close encounter. The left panel shows the Monte Carlo samples as blue scatter points. Ellipses were calculated using 4 methods: Analytic STM (red dashed line), Central finite difference STM (gray dotted line), Monte Carlo sampling (blue dashed-dotted line), and the new unscented transform STM (green solid line). All ellipses in the right panel have been rotated such that the major axis of the Monte Carlo ellipse is on the vertical to better help in distinguishing between the ellipses. The analytic, unscented STM, and central difference STMs are indistinguishable because they overlap. The ellipses have been centered at the Monte Carlo mean. Note the different scales on the axes

Figure 5 shows the $3\sigma$ uncertainty ellipses of the B-plane coordinates for Bennu during the Earth close encounter on 22 September 2080. The close encounter occurs at a distance of $2.3 \times 10^{6}\,\hbox {km}$. The dimensions of the Monte Carlo uncertainty ellipse are 6852.5 and 1.1 km for the semimajor and semiminor axes, respectively. The ellipses were computed using four different techniques. As expected from the previous tests, since the nonlinearities are small in 2080, there is excellent agreement between all four ellipses. In terms of quantifiable differences, the central difference partials were the worst, with a difference of 0.70% in the semimajor axis and $-$1.64% in the semiminor axis compared to the covariance of the Monte Carlo samples. In contrast, the analytic STMs had a difference of 0.56 and $-$1.64% in the semimajor and semiminor axes, respectively. Finally, the unscented transform STMs had a difference of 0.56 and $-$1.65% in the semimajor and semiminor axes, respectively. The differences in orientation angles were too small to be important (less than 0.005% for all STMs). This shows that the unscented transform STMs are just as accurate as Monte Carlo sampling for the 2080 encounter. The nonlinearities in this close approach are small; the Monte Carlo samples are symmetric about the abscissa. However, after this encounter, the uncertainties in the B-plane coordinates increase significantly (while remaining fully predictable until September 2135).

**Fig. 6:**
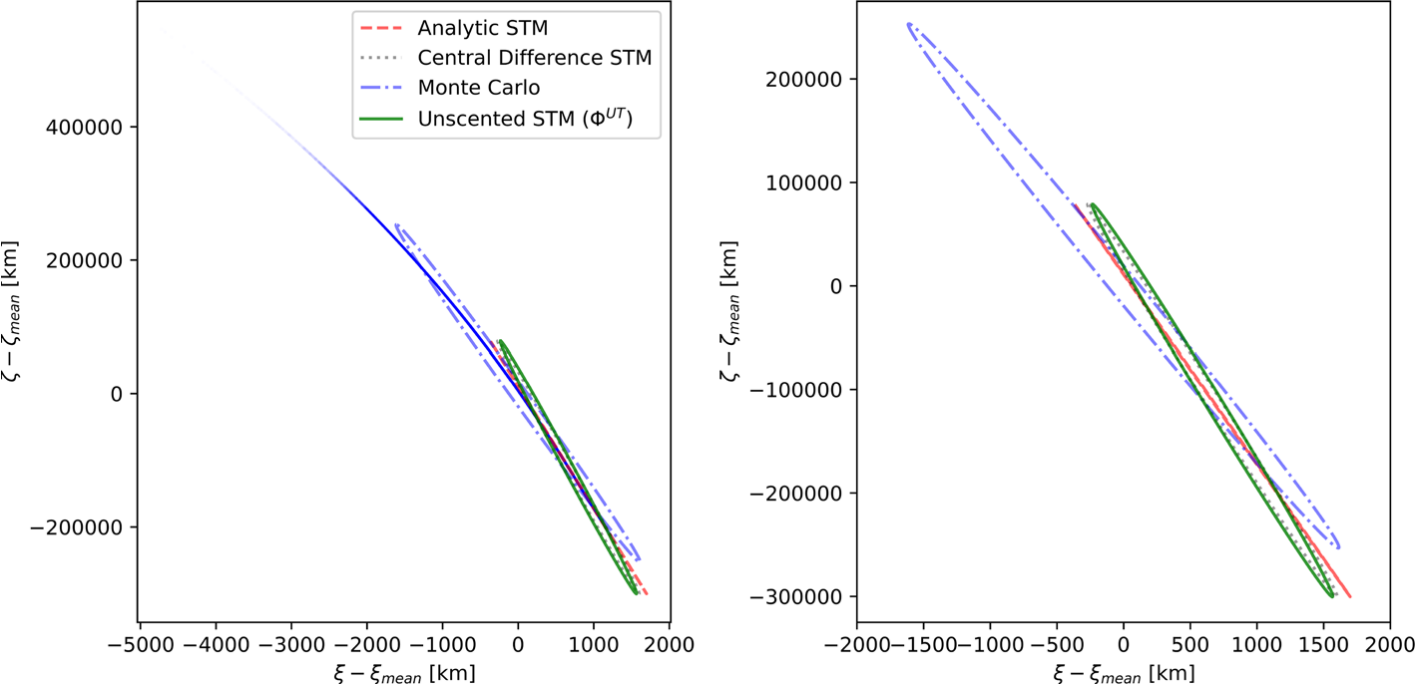
3$\sigma$ ellipses of the B-plane uncertainties for Bennu during the 2135 Earth close encounter. The left panel shows the Monte Carlo samples as blue scatter points. Ellipses were calculated using 4 methods: Analytic STM (red dashed line), Central finite difference STM (gray dotted line), Monte Carlo sampling with 10$^6$ samples (blue dashed-dotted line), and the new unscented transform STM (green solid line). The limits in the right panel have been chosen to better help in distinguishing between the ellipses. The ellipses have been centered at the Monte Carlo mean. Note the different scales on the axes

Figure 6 shows the $3\sigma$ uncertainty ellipses of the B-plane coordinates for Bennu during the Earth close encounter on 25 September 2135. The close encounter nominally occurs at a distance of $3.2 \times 10^{5}\,\hbox {km}$. The dimensions of the Monte Carlo uncertainty ellipse are 84 429.5 and 41.2 km for the semimajor and semiminor axes, respectively. The ellipses were computed using the same four techniques and follow the same color and line styles as Fig. 5. Due to the large amount of time (more than 1 century) between the observation data and the close encounter, as well as the deep planetary encounters that Bennu experiences in that time, there are significant nonlinearities in this close approach. This is evidenced by the lack of any symmetry in the Monte Carlo points around the origin in Fig. 6. As a result, there are some significant differences in the ellipses.

The analytic partials were relatively the worst for this encounter, with differences of $-$25.21% in the semimajor axis and $-$97.29% in the semiminor axis compared to the covariance of the Monte Carlo samples. This is because the accuracy of the analytic STM starts to worsen in the presence of significant nonlinearities. In contrast, the central difference STMs had a difference of $-$25.04 and $-$48.01% in the semimajor and semiminor axes, respectively. Finally, the unscented transform STM performed the best and had a difference of $-$25.14 and 42.02% in the semimajor and semiminor axes, respectively. As far as the orientation angle is concerned, the unscented transform STMs were off by about 0.10%, the central difference partials were off by about 0.09%, and the analytic STMs were off by about 0.06%.

These results show that the unscented transform STMs can be used in the presence of stiffness in the dynamics and that they can outperform analytic STMs and match second-order central finite difference algorithms at the same computational cost. More importantly, they do not require the manual selection of the perturbation needed to compute the central difference STMs.

Once again, the unscented transform STM was used to map covariances, this time for Bennu’s close approach B-plane coordinates. In this scenario, the use of the unscented transform STMs enabled this analysis. This is because not all sigma point formulations guarantee a positive semi-definite posterior covariance matrix (Julier et al. 2000; Julier 2002). For instance, if using the unscented transform to map the 2135 Bennu–Earth close approach, the uncertainty in the time of close approach can be negative, which is nonsensical^3^. However, the method of using the unscented transform STM at the time of close approach and then the analytic B-plane partials to map the covariance alleviates this problem. This shows that the unscented transform STMs allow one to perform more complex analyses where other methods may fail or have debilitatingly poor performance.

## Conclusions

A new method for computing the state transition matrix (STM) of a nonlinear system using the unscented transform was presented in this paper. The method uses the sigma points as perturbations to the nominal state and maps these perturbations to the desired final time. The STM is then computed using the positively and negatively perturbed sigma points. The unscented transform STMs were validated using three different applications: a two-body problem, an EDL flight mechanics problem, and close encounters of the asteroid (101955) Bennu with the Earth.

In all three applications, these new STMs had accuracy similar to or better than that of traditional analytic STMs. In the last of the three applications, the unscented transform STMs were also shown to outperform analytic STMs while matching central finite difference STMs, which is the closest existing technique to this new method. This application also demonstrated the use of these unscented transform STMs in the presence of significant stiffness in the dynamics, where other methods fail. One drawback of this new technique is that it requires the calculation of sigma points, which in turn require an initial covariance matrix. However, this is not a critical issue. If a covariance matrix is not available under the natural course of solving a problem, it must be assumed. In such a scenario, the unscented STMs computed from assumed standard deviations behave similarly to central difference STMs with the corresponding finite difference steps.

Unscented transform STMs are a powerful tool that can be used to map the covariance of the state while providing insight into the dynamical sensitivities of the system. Furthermore, a single unscented STM can map many covariance matrices (similar to analytic or finite difference STMs) without the need to re-generate the sigma points for each covariance matrix. Future work should involve applying the unscented transform STMs to operational problems in celestial and spaceflight mechanics, such as asteroid or comet orbit determination for understanding their impact hazard.

## Footnotes

1 https://nssdc.gsfc.nasa.gov/planetary/factsheet/marsfact.html

2 GRSS is publicly available at https://github.com/rahil-makadia/grss

3 This occurs when sigma points are generated according to Eqs. 1 and 2 with $\kappa = 3-n$, as recommended for Gaussian initial distributions (e.g., Bennu’s orbit solution) by Julier and Uhlmann (1997). For orbit propagation, $n=6$ and therefore $\kappa < 0$. This leads to negative weights, and therefore negative posterior uncertainties when reconstructing the posterior distribution using Eq. 3.
